# The vaginal microflora in relation to gingivitis

**DOI:** 10.1186/1471-2334-9-6

**Published:** 2009-01-22

**Authors:** Rutger Persson, Jane Hitti, Rita Verhelst, Mario Vaneechoutte, Rigmor Persson, Regula Hirschi, Marianne Weibel, Marilynn Rothen, Marleen Temmerman, Kathleen Paul, David Eschenbach

**Affiliations:** 1 Department of Periodontology, Div of Oral Microbiology, University of Berne, Berne, Switzerland; 2 Department of Periodontics, University of Washington, Seattle, WA, USA; 3 Department of Oral Medicine, University of Washington, Seattle, WA, USA; 4 Department of Obstetrics & Gynecology, University of Washington, Seattle, WA, USA; 5 Department of Clinical Chemistry, Microbiology and Immunology, Ghent University, Ghent, Belgium; 6 Regional Clinical Dental Research Center (RCDRC), University of Washington, Seattle, WA, USA; 7 Department of Obstetrics and Gynaecology, Faculty of Medicine and Health Sciences, Ghent University, Ghent, Belgium

## Abstract

**Background:**

Gingivitis has been linked to adverse pregnancy outcome (APO). Bacterial vaginosis (BV) has been associated with APO. We assessed if bacterial counts in BV is associated with gingivitis suggesting a systemic infectious susceptibilty.

**Methods:**

Vaginal samples were collected from 180 women (mean age 29.4 years, SD ± 6.8, range: 18 to 46), and at least six months after delivery, and assessed by semi-quantitative DNA-DNA checkerboard hybridization assay (74 bacterial species). BV was defined by Gram stain (Nugent criteria). Gingivitis was defined as bleeding on probing at ≥ 20% of tooth sites.

**Results:**

A Nugent score of 0–3 (normal vaginal microflora) was found in 83 women (46.1%), and a score of > 7 (BV) in 49 women (27.2%). Gingivitis was diagnosed in 114 women (63.3%). Women with a diagnosis of BV were more likely to have gingivitis (p = 0.01). Independent of gingival conditions, vaginal bacterial counts were higher (p < 0.001) for 38/74 species in BV+ in comparison to BV- women. Counts of four lactobacilli species were higher in BV- women (p < 0.001). Independent of BV diagnosis, women with gingivitis had higher counts of *Prevotella bivia *(p < 0.001), and *Prevotella disiens *(p < 0.001). *P. bivia, P. disiens, M. curtisii *and *M. mulieris *(all at the p < 0.01 level) were found at higher levels in the BV+/G+ group than in the BV+/G- group. The sum of bacterial load (74 species) was higher in the BV+/G+ group than in the BV+/G- group (p < 0.05). The highest odds ratio for the presence of bacteria in vaginal samples (> 1.0 × 10^4 ^cells) and a diagnosis of gingivitis was 3.9 for *P. bivia *(95% CI 1.5–5.7, p < 0.001) and 3.6 for *P. disiens *(95%CI: 1.8–7.5, p < 0.001), and a diagnosis of BV for *P. bivia *(odds ratio: 5.3, 95%CI: 2.6 to 10.4, p < 0.001) and *P. disiens *(odds ratio: 4.4, 95% CI: 2.2 to 8.8, p < 0.001).

**Conclusion:**

Higher vaginal bacterial counts can be found in women with BV and gingivitis in comparison to women with BV but not gingivitis. *P. bivia *and *P. disiens *may be of specific significance in a relationship between vaginal and gingival infections.

## Background

Adverse preterm outcomes occur in approximately 10% of all pregnancies [1]. It remains a major source of neonatal morbidity and mortality. The prevalence of periodontitis in women of childbearing age is unknown. Gingivitis is a reversible inflammatory condition of keratinized and non-keratinized gum tissues surrounding the teeth. Periodontitis is a non-reversible inflammatory condition that also includes loss of alveolar bone and other tooth supporting structures. Infection with a diverse microflora is the etiology of both these conditions. The association between gingivitis or periodontitis and an increased risk of preterm birth remains a matter of dispute. Several recent studies support the hypothesis that periodontal infectious disease is a risk factor for adverse pregnancy outcomes [2-8]. One hypothesized mechanism is that inflammation may upregulate the inflammatory response in anatomically distinct locations such as the uterus and the amniotic cavity [7-9].

Bacterial vaginosis (BV), a condition characterized by decreased vaginal lactobacilli and increased anaerobic bacteria, has been associated with an increased risk of preterm birth [10,11]. The abnormal microflora typical of BV overlaps considerably with bacterial species known to be associated with periodontal disease. For example, *Prevotella bivia *and *Porphyromonas *sp. have been associated with BV [12], whereas *Prevotella intermedia *and *Porphyromonas gingivalis *have been associated with periodontal disease [13,14]. Higher counts of colony forming units of *P. gingivalis *in subgingival samples have also been observed in women who subsequently delivered prematurely [8,15]. Despite such findings, the biological relationship between oral and vaginal infections has not been extensively studied.

The purpose of the present study was to characterize the bacterial species in vaginal samples from women of childbearing age in relation to clinical evidence of gingival inflammation (gingivitis) and bacterial vaginosis. We hypothesized that the vaginal microflora differed between women with or without overt clinical evidence of gingivitis. We also hypothesized a co-occurrence of BV and gingivitis.

## Methods

The Human Research Review Board of the Washington State Department of Health approved the study. All subjects signed informed written consent as required by the IRB. The study cohort included parous women with no known systemic disease, who were recruited based on a previous history of early preterm delivery (20–34 weeks gestation) or term delivery (≥ 37 weeks gestation). A preterm birth occurred among 17 (9.2%) of the women participating in the present study. All women had delivered at least 6 months prior to study entry and microbiological sampling.

The women had a gynecological examination with collection of vaginal by insertion of a Dacron swab into the vaginal vault. One swab was used to prepare an air-dried slide for Gram stain for BV diagnosis according to the Nugent criteria [11]. A second swab tip was placed in a cryovial eluted in 0.9 ml phosphate buffered saline and stored at -80°C until transported on dry ice by express courier to the Oral Microbiology Laboratory at the University of Berne, Switzerland, for analysis of microbial content.

Women also had a standard periodontal examination at the Regional Clinical Dental Research Center (RCDRC), School of Dentistry at the University of Washington, Seattle, WA. Gingivitis was defined as having ≥ 20% of gingival sites surfaces (six examined per tooth) with bleeding on probing (BOP). This cutoff level is considered a useful criterion to establish gingivitis and is used as a basic principle in the standard practice of periodontal care. Clinical probing pocket depths around all teeth and intra-oral radiographs were also assessed to define if the women also had a diagnosis of periodontitis (alveolar bone loss and evidence of increased probing pocket depth/clinical attachment loss).

A total of 180 women who had given birth at least six months prior to enrollment were included in this analysis. Their mean age was 29.4 years (SD ± 6.8, range: 18 to 46). The racial groups were as follows: 60 Caucasians (32.6%), 82 African-Americans (44.6%), 9 Native Americans (4.9%), and 33 of other races (17.9%).

### Microbiological processing

At the microbiology laboratory, 300 μl Tris EDTA buffer (10 mM Tris-HCL, 1.0 mM EDTA, pH 7.6) was added to each vial with a vaginal swab, allowed to stand for 10 minutes, and was then sonicated for 10 seconds. Subsequently, 200 μl of freshly made 0.5 M NaOH was added to each vial and the swab was removed before freezing the sample. Samples were then processed within three months. Before processing, the samples were diluted fourfold with Tris EDTA buffer and aliquoted into two vials. These were processed by the checkerboard DNA-DNA hybridization method as described in detail elsewhere [16-19]. DNA probes used in the checkerboard DNA-DNA format provide a useful tool for the enumeration of bacterial species in microbiologically complex systems [17]. One vial was used to check for the presence of the species of which probes were present on the first panel, the other vial for the species of the second panel (Table 1). The information was digitized and analyzed by the software program ImageQuant (Amersham Pharmacia, Piscataway, NJ) allowing comparison of signal intensities against standard lanes containing DNA extracted from 10^5 ^and 10^6 ^bacterial cells in the appropriate checkerboard slot for all species. Routine laboratory assessment of the reproducibility suggested a very high level of reliability varying within species by 1–2%. Signals were converted to semi-quantitative counts by comparison with these standards. For dichotomous analysis of the data, a signal strength of ≥ 1 × 10^4 ^bacterial cells was considered as positive. The species that were studied are listed in Table 1. The species listed in Panel 1 are commonly assessed by the checkerboard DNA-DNA hybridization method in studies of bacteria associated with periodontitis [19,20]. These were either part of the original microbiological laboratory library of species [19,20] or had been provided by the Department of Clinical Chemistry, Microbiology and Immunology at the University of Ghent, Belgium (UGent). Some species or DNA had been purchased from LGC Promochem, Molsheim, France. The identification of species from the University of Ghent has been described elsewhere [21-24]. Thus the bacteria listed in Panel 2 are commonly assessed in studies of BV.

**Table 1:** Bacterial species and subspecies included in the DNA-DNA checkerboard assay

Species Panel 1	Collection*	Species Panel 2	Collection*
*1a. Aggregatibacter actinomycetemcomitans (a)*	ATCC 29523	*1. Actinomyces neuii*	GUH 550898
*1b. Aggregatibacter actinomycetemcomitans (Y4)*	ATCC 43718	*2. Aerococcus christensenii*	GUH 070938
*2. Actinomyces israelii*	ATCC 12102	*3. Anaerococcus vaginalis*	GUH 290486
*3. Actinomyces naeslundii (type I + II)*	ATCC 43146	*4. Atopobium parvulum*	GUH 160323
*4. Actinomyces odontolyticum*	ATCC 17929	*5. Atopobium vaginae*	GUH 010535
*5. Campylobacter gracilis*	ATCC 33236	*6. Bacteroides ureolyticus*	GUH 080189
*6. Campylobacter rectus*	ATCC 33238	*7. Bifidobacterium biavatii*	GUH 071026
*7. Campylobacter showae*	ATCC 51146	*8. Bifidobacterium bifidum*	GUH 070962
*8. Capnocytophaga gingivalis*	ATCC 33612	*9. Bifidobacterium breve*	GUH 080484
*9. Capnocytophaga ochraceae*	ATCC 33596	*10. Bifidobacterium longum*	GUH 180689
*10. Capnocytophaga sputigena*	ATCC 33612	*11. Corynebacterium aurimucosum*	GUH 450453
*11. Eikenella corrodens*	ATCC 23834	*12. Corynebacterium nigricans*	GUH 071035
*12. Eubacterium saburreum*	ATCC 33271	*13. Dialister *sp.	GUH071045
*13a. Fusobacterium nucleatum *subsp. *Nucleatum*	ATCC 25586	*14a. Enterococcus faecalis*	GUH170812
*13b. Fusobacterium nucleatum *subsp. *Polymorphum*	ATCC 10953	*14b. Enterococcus faecalis*	ATCC 29212
*13c. Fusobacterium nucleatum *subsp.*Naviforme*	ATCC 49256	*15. Escherichia coli*	GUH 070903
*14. Fusobacterium periodonticum*	ATCC 33693	*16. Gardnerella vaginalis*	GUH 080585
*15. Lactobacillus acidophilus*	ATCC 11975	*17. Haemophilus influenzae*	ATCC 49247
*16. Leptotrichia buccalis*	ATCC 14201	*18. Helicobacter pylori*	ATCC 43504
*17. Parvimonas micra*	ATCC 19696	*19. Lactobacillus crispatus*	GUH 160342
*18. Neisseria mucosa*	ATCC 33270	*20. Lactobacillus gasseri*	GUH 170856
*19. Prevotella intermedia*	ATCC 25611	*21. Lactobacillus iners*	GUH 160334
*20. Prevotella melaninogenica*	ATCC 25845	*22. Lactobacillus jensenii*	GUH 160339
*21. Prevotella nigrescens*	ATCC 33563	*23. Lactobacillus vaginalis*	GUH 0780928
*22. Porphyromonas gingivalis*	ATCC 33277	*24. Mobiluncus curtisii*	GUH 070927
*23. Propionibacterium acnes (type I+II)*	ATCC11827/28	*25. Mobiluncus mulieris*	GUH 070926
*24. Selenomonas noxia*	ATCC 43541	*26. Peptoniphilus *sp.	GUH 550970
*25. Staphylococcus aureus*	ATCC 25923	*27. Porphyromonas endodontalis*	ATCC35406
*26. Streptococcus anginosus*	ATCC 33397	*28. Peptostreptococcus anaerobius*	GUH 160362
*27. Streptococcus constellatus*	ATCC 27823 (M32b)	*29. Prevotella bivia*	GUH 450429
*28. Streptococcus gordonii*	ATCC 10558	*30. Prevotella disiens*	GUH 190184
*29. Streptococcus intermedius*	ATCC 27335	*31. Prevotella mirabilis*	GUH 070918
*30. Streptococcus mitis*	ATCC 49456	*32. Pseudomonas aeruginosa*	ATCC 33467
*31. Streptococcus oralis*	ATCC 35037	*33a. Staphylococcus aureus *(yellow)	GUH 070921
*32. Streptococcus sanguinis*	ATCC 10556	*33b. Staphylococcus aureus *(white)	GUH 070922
*33. Streptococcus mutans*	ATCC 25175	*34. Staphylococcus epidermidis*	GUH 130381
*34. Tannerella forsythia*	ATCC 43037 (338)	*35. Staphylococcus haemolyticus*	GUH071047
*35. Treponema denticola*	ATCC 35405	*36. Streptococcus agalactiae*	GUH 230282
*36. Treponema socranskii*	D40DR2	*37. Varibaculum cambriense*	GUH 070917
*37. Veillonella parvula*	ATCC 10790		

Legend: * ATCC: American Type Culture Collection; D: sample from Forsyth Institute, Massachusetts; GUH: Ghent University Hospital Collection, Ghent, Belgium

### Statistical methods

We used One-way ANOVA (Bonferonni post-hoc test) and non-parametric Mann-Whitney U tests, and Kruskal-Wallis ANOVA to assess differences in the quantity of each bacterial species by defined group. Adjustment for multiple comparisons was made and a statistically significant difference was defined by p < 0.001. P-values < 0.01 and < 0.05 were considered as trends of difference. Mantel-Haenszel common odds ratios, sensitivity, and specificity estimations were calculated in order to assess the predictive utility of each vaginal bacterial species in diagnosing gingivitis. The SPSS statistical software 16.0 for MAC OS X was used for the analysis (SPSS Inc., Chicago, IL).

## Results

A Nugent score of 0–3, considered as indicative for normal vaginal microflora without signs of inflammation, was found in 83 women (46.1%), and a score of > 7, i.e. clear BV was present in 49 women (27.2%). A total of 38 women (21.1%) had both BV and gingivitis, 54 (30.0%) showed no evidence of either BV or gingivitis, 17 (9.4%) had BV but not gingivitis, and 71 (39.4%) had gingivitis but not BV. Women with a diagnosis of BV had a higher proportion of tooth surfaces with evidence of gingivitis (p = 0.007).

### The vaginal microflora of women in relation to a diagnosis of bacterial vaginosis, and independent of gingival conditions

The bacterial species assessed are identified in Table 1. The presence of streptococci, staphylococci and enterococci studied did not differ by BV status. In vaginal samples from women with BV, but independent of gingival status significantly higher bacterial loads (p < 0.001) were observed for the following 38/74 species: *A. actinomycetemcomitans (Y4,), A. israelii, A. naeslundi, A. neuii, A. odontolyticus, A. christensenii, B. biavatii, B. longum, B. ureolyticus, C. gingivalis, C. aurimucosum, C. ochraceae, C. sputigena, C. gracilis, C. rectus, C. showae, E. coli, E. corrodens, F. nucl. naviforme, F. nucl. nucleatum, F. nucl. polymorphum, F. periodonticum, H. influenzae, M. curtisii, M. mulieris, P. micra, P. gingivalis, P. bivia, P. disiens, P. intermedia, P. melaninogenica, P. nigrescens, P. acnes, P. aeruginosa, T. forsythia, S. noxia, T. socranskii *and *V. cambriense*. However *, L. crispatus*, *L. gasseri*, *L. iners*, and *L. vaginalis *were found at higher counts at sites without BV (p < 0.001).

### The relationship between the vaginal microflora and gingivitis

Significantly higher vaginal bacterial counts (p < 0.001) were found for 49/74 species (23 from panel 1 and 26 from panel 2) in BV+ women with a concurrent diagnosis of gingivitis as compared to women who neither had BV nor gingivitis. At the p < 0.001 level this included in addition to those reported above the following species: *A. vaginae, B. brevis, C. nigricans, Dialister sp., E. saburreum, L. buccalis, N. mucosa, Petoniphilus sp., P. nigrescens, P. anaerobius*, and *V. parvula*.

Table 2 presents the prevalence rates of bacterial species collected from vaginal samples at the > 1 × 10^4 ^detection level demonstrating statistically significant differences by gingival status but independent of BV diagnosis. For 2 of the 74 species tested, *P. bivia *and *P. disiens*, higher bacterial counts were observed in subjects with gingivitis (Mann-Whitney U-test, p < 0.001). Trends of differences at the p < 0.01 level were also observed for *B. ureolyticus*, *M. curtisii*, *M. mulieris*, and *P. aeruginosa*, as well as at the p < 0.05 for *G. vaginalis, P. intermedia, P. nigrescens*, and *V. cambriense*. The sensitivity, specificity and odds ratio characteristics of the predictive values for the 6 species with statistically significant odds distinguishing gingival status are presented in Table 3. The remaining 4 species identified in Table 2 failed to qualify. Thus, when *P. bivia *was present in the vaginal samples, the odds ratio for gingivitis was 3.9 (95% CI 1.5–5.7, p < 0.001). When *P. disiens *was present in the vaginal samples, the odds ratio for a diagnosis of gingivitis was 3.6 (95%CI: 1.8–7.5, p < 0.001). The corresponding odds ratio for a diagnosis of BV was 5.3 for *P. bivia *(95%CI: 2.6 to 10.4, p < 0.001) and 4.4 for *P. disiens *(95% CI: 2.2 to 8.8, p < 0.001).

**Table 2:** Prevalence of vaginal bacterial species (cut off level: > 1 × 10^4 ^cells) for which a significant difference was observed by gingivitis status

Species	No gingivitis (n = 64)	Gingivitis (n = 116)	p-value
***Prevotella bivia***	**21.4**	**43.8**	**< 0.001**

***Prevotella disiens***	**15.7**	**40.2**	**< 0.001**

*Bacteroides ureolyticus*	7.1	17.0	< 0.01
*Mobiluncus curtisii*	15.7	34.8	< 0.01
*Mobiluncus mulieris*	7.9	24.1	< 0.01
*Pseudomonas aeruginosa*	10.0	20.5	< 0.01
*Gardnerella vaginalis*	60.0	67.2	< 0.05
*Prevotella intermedia*	60.0	69.2	< 0.05
*Prevotella nigrescens*	10.8	24.3	< 0.05
*Varibaculum cambriense*	21.4	33.0	< 0.05

Legend: Expressed as percentages.

**Table 3:** The predictive value of the presence (cut off level: 1 × 10^4 ^cells) of *P. bivia, P. disiens, M. curtisii, M. mulieris, B. ureolyticus*, and *V. cambriense *in vaginal samples for the diagnosis of gingivitis (20% cutoff level)

Microorganism	Sensitivity	Specificity	Odds ratio	95% CI	P-value
*Prevotella bivia*	0.18	0.97	3.9	1.7 to 33.8	< 0.001
*Prevotella disiens*	0.57	0.83	3.6	1.8 to 7.1	< 0.001
*Mobiluncus curtisii*	0.37	0.83	3.8	1.4 to 5.8	< 0.01
*Mobiluncus mulieris*	0.26	0.93	4.6	1.8 to 12.6	< 0.01
*Bacteroides ureolyticus*	0.21	0.93	3.3	1.2 to 9.1	< 0.02
*Varibaculum cambriense*	0.38	0.77	2.0	1.0 to 4.1	< 0.05

### Differences in bacterial levels by differentiation between subjects with any of four BV and gingivitis diagnostic combinations

**Figure 1:**
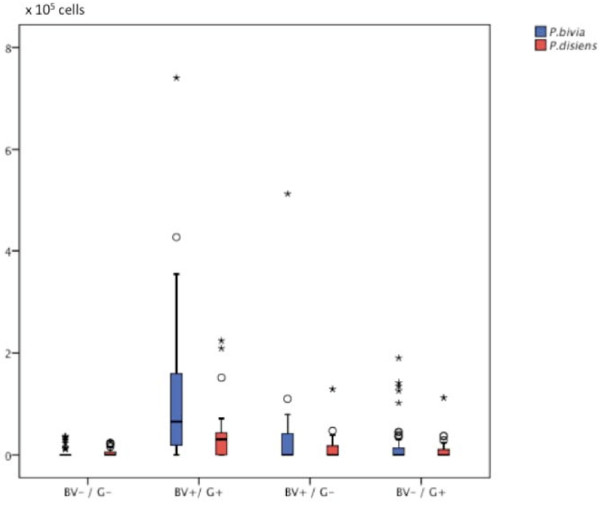
**Boxplot diagram demonstrating differences in vaginal load for P. bivia and P. disiens by combined diagnostic criteria (Bacterial vaginosis neg and gingivitis neg (BV-/G-), Bacterial vaginosis pos. and Gingivitis pos. (BV+/G+), Bacterial vaginosis pos. and Gingivitis neg. (BV+/G-), and Bacterial vaginosis neg. and Gingivitis pos. (BV-/G+)**. (* = extreme outliers, ° = outlier values).

The distributions of *P. bivia *and *P. disiens *in vaginal samples for the four different populations according to combination of vaginal and gingival microflora (BV+/G+, BV-/G-, BV+/G- and BV-/G+) are presented in a boxplot diagram (Figure 1). Analysis by Kruskal-Wallis ANOVA identified differences in bacterial levels at the p < 0.001 level by the combined vaginal and periodontal diagnostic criteria for 49/74 species identified in vaginal samples between BV+/G+ and BV-/G- women and at the p < 0.001 level for the following species: *A. actinomycetemcomitans *(serotype Y4), *A. actinomycetemcomitans *(serotype b), *A. israelii, A. naeslundii, A. neuii, A. christensenii, A. vaginae, A. odontolyticus, B. ureolyticus, B. biavatii, B. breve, B. longum, C. nigricans, C. aurimucosum, C. gingivalis, C. gracilis, C. ochracea, C. rectus, C. showae, Dialister *sp. *E. coli, E. corrodens, E. saburreum, F. nucl. nucleatum, F. nucl. polymorphum, F. nucl. naviforme, F. periodonticum, H. influenzae, L. buccalis, M. curtisii, M. mulieris, Peptoniphilus *sp., *N. mucosa, P. aeruginosa, P. micra, P. anaerobius, P. mirabilis, P. bivia, P. disiens, P. intermedia, P. melaninogenica, P. nigrescens, P. gingivalis, P. acnes, S. noxia, T. forsythia, T. socranskii, V. cambriense*, and *V. parvula*.

Specifically, higher counts in vaginal samples were found in women in the BV+/G+ group in comparison to the BV+/G- group for *P. bivia, P. disiens, M. curtisii*, and *M. mulieris *(all at the p < 0.01 level). Further analysis demonstrated that the sum of bacterial load including all 74 species studies was higher in the BV+/G+ group than in the BV+/G- group (p < 0.05), but was also higher than in separate comparisons with the two other possible diagnostic combinations (p < 0.01).

## Discussion

The checkerboard DNA-DNA hybridization method has been demonstrated to be useful in studies of changes in vaginal microflora during pregnancy [25,26]. Checkerboard DNA-DNA hybridization is also commonly used in studies on the oral microflora [i.e. [13,14,16-18,20]], and has also been used to assess the microflora in synovial fluid of subjects with rheumatoid arthritis [27]. Studies of the bacterial colonization of the oral cavity have shown that *Actinomyces *sp. [28], *Fusobacterium *spp. [29], *Prevotella *sp. [30], *Capnocytophaga *sp. [31] and *T. forsythia *[32] are associated with gingivitis.

In accordance with the study by Boggess *et al*. [25], who also using DNA-DNA hybridization in the assessment of vaginal species the present study identified that *B. ureolyticus *and *M. curtisii *were commonly found in vaginal samples. We also indentified that, in women with BV and gingivitis and in comparison with those with BV but not gingivitis, the vaginal samples demonstrated significantly higher counts of bacteria commonly associated with periodontal disease including: *A. actinomycetemcomitans *(Y4), *Fusobacterium *sp., *P. micra, P. intermeda P. gingivalis*, and *T. forsythia*. The role of these bacteria as potential infectious etiological factors in adverse pregnancy outcomes in the context of oral/gingival infection needs to be further explored as also indicated by others [8,15]. The fact that some bacterial species were found at higher counts in the vaginal samples of women with BV and gingivitis than among those only with BV may be an important observation suggesting that having gingivitis has an impact on the bacterial load in women with BV.

The prevalence of gingivitis in our study population (62%) was higher than the gingivitis prevalence of 48% reported in NHANES III (National Health and Nutrition Examination Survey) [33]. This may be explained by the ethnic distribution and inclusion of many women with low socio-economic status [34]. Studies that have associated periodontal disease with a risk of preterm birth have used a variety of periodontal diagnostic criteria against which the risk of preterm birth has been considered. One of the problems is that periodontitis usually does not present with unequivocal clinical evidence of disease until subjects are at about age 40. Gingivitis is common in younger age groups. The inflammatory response in gingivitis is primarily a cellular immune response [35]. Data from one study suggest that treatment of gingivitis in pregnant women significantly reduced the risk of preterm birth [36]. Due to the fact that only 17 of the women with a high risk for a preterm birth complication in the present study delivered preterm, a statistical analysis of the data based on delivery status was not performed.

This is the first study to demonstrate a link between vaginal bacteria and gingivitis. Others have suggested that the hematogenous route allows the spread of opportunistic pathogens from one location to another [35]. It is also plausible that opportunistic bacteria take advantage of host specific factors and colonize wherever growth conditions are suitable. The oral and vaginal environments may provide such similar colonization and growth conditions in a susceptible host. Further studies are warranted to explore the relationship between oral and vaginal microflora and infections. Further studies are warranted to explore the relationship between the oral and vaginal infection patterns and how this may explain why periodontal disease (including gingivitis) may be a potential adjunct cause of adverse pregnancy outcomes [2-8,15,36].

In a recent periodontal intervention study of pregnant women with periodontitis, the data failed to demonstrate that periodontal intervention had an impact on pregnancy outcomes [37]. Treatment was also restricted to mechanical subgingival debridement and may not have affected bacterial presence in relation to persistent gingivitis, or reduced gingivitis to levels < 20% used as criteria in the present study. Although the survival rate of premature neonates has improved greatly in developed countries, the prevalence of preterm birth rates have not declined and various intervention studies to control for BV and other risk factors have not been successful [38]. Thus, significantly reducing preterm births may require a broader approach to care not limited to vaginal conditions but also include oral infections such as gingivitis.

## Conclusion

• Women with a diagnosis of BV have a higher proportion of tooth surfaces with evidence of gingivitis

• Women with BV and gingivitis and in comparison to those without BV and gingivitis have higher vaginal bacterial counts including bacteria commonly associated with both BV periodontal disease than women with BV but without a diagnosis of gingivitis

• *P. bivia*, and *P. disiens*, may be of importance in women with both BV and gingivitis.

## Competing interests

The authors declare that they have no competing interests.

## Authors' contributions

GRP, JH, DE, MV, MT and RV designed the study. MR collected clinical dental data and JH was in charge of medical examinations. REP analyzed the radiographs and defined the periodontal diagnosis. KP coordinated the study and established the clinical database (medical and dental). MW and RH processed the microbiological material and developed the probes. RV, MT and MV provided and confirmed the accuracy of the bacteria. GRP was responsible for the data analysis. All co-authors contributed to the preparation of the manuscript.

## Pre-publication history

The pre-publication history for this paper can be accessed here:

http://www.biomedcentral.com/1471-2334/9/6/prepub
